# Structural basis of sialidase in complex with geranylated flavonoids as potent natural inhibitors

**DOI:** 10.1107/S1399004714002971

**Published:** 2014-04-30

**Authors:** Youngjin Lee, Young Bae Ryu, Hyung-Seop Youn, Jung Keun Cho, Young Min Kim, Ji-Young Park, Woo Song Lee, Ki Hun Park, Soo Hyun Eom

**Affiliations:** a School of Life Sciences, Gwangju Institute of Science and Technology (GIST), Buk-gu, Gwangju 500-712, Republic of Korea; b Steitz Center for Structural Biology, Gwangju Institute of Science and Technology (GIST), Buk-gu, Gwangju 500-712, Republic of Korea; c Department of Chemistry, Gwangju Institute of Science and Technology (GIST), Buk-gu, Gwangju 500-712, Republic of Korea; d Infection Control Research Center, Korea Research Institute of Bioscience and Biotechnology, Jeongeup 580-185, Republic of Korea; e Division of Applied Life Science (BK21 Program, IALS), Graduate School of Gyeongsang National University, Jinju 660-701, Republic of Korea

**Keywords:** sialidase, NanI, geranylated flavonoid, diplacone, sialidase inhibitor

## Abstract

Sialidase catalyzes the removal of a terminal sialic acid from glycoconjugates and plays a pivotal role in nutrition, cellular interactions and pathogenesis mediating various infectious diseases including cholera, influenza and sepsis. An array of antiviral sialidase agents have been developed and are commercially available, such as zanamivir and oseltamivir for treating influenza. However, the development of bacterial sialidase inhibitors has been much less successful. Here, natural polyphenolic geranylated flavonoids which show significant inhibitory effects against *Cp*-NanI, a sialidase from *Clostridium perfringens*, are reported. This bacterium causes various gastrointestinal diseases. The crystal structure of the *Cp*-NanI catalytic domain in complex with the best inhibitor, diplacone, is also presented. This structure explains how diplacone generates a stable enzyme–inhibitor complex. These results provide a structural framework for understanding the interaction between sialidase and natural flavonoids, which are promising scaffolds on which to discover new anti-sialidase agents.

## Abstract 2

The crystal structure of sialidase from *C. perfringens*, a pathogenic bacterium causing various gastrointestinal diseases, was determined in complex with a potent natural polyphenolic geranylated flavonoid-based inhibitor. The complex structure and comparative kinetic studies revealed that the geranyl group and C3′ hydroxyl group of the flavonoid backbone contribute to inhibition of the bacterial sialidase and generation of the stable enzyme–inhibitor complex.

## 1. Introduction   

Viral and bacterial sialidases have been focused on as drug targets for the treatment of human infections (Soong *et al.*, 2006 ▶; Memoli *et al.*, 2008 ▶). Viral sialidases are critically required for viral propagation because they are required for release from the host cell (Kim *et al.*, 2013 ▶). Drug discovery of inhibitors targeting sialidase has focused on either synthetic compounds through random screening or substrate (*N*-acetylneuraminic acid) mimics. However, these studies have mainly been targeted against sialidase from the influenza virus. There are several inhibitors that mimic the substrate, including zanamivir (Relenza), oseltamivir (Tamiflu; a cyclohexene derivative), peramivir (a cyclopentane-based inhibitor) and A-315675 (a pyrrolidine-based inhibitor; Kim *et al.*, 2013 ▶). All of these compounds are based on 2-deoxy-2,3-dehydro-*N*-acetylneuraminic acid (Neu5Ac2en), a putative transition-state analogue. At present, the existence of drug-resistant strains against zanamivir (Gubareva *et al.*, 1998 ▶), oseltamivir (Gubareva *et al.*, 2001 ▶) and other antivirals (Tambić Andrasević, 2004 ▶) have spurred on the need to discover innovative antiviral agents.

On the other hand, pathogenic bacteria having resistance to pre-existing drugs occasionally cause fatal infectious diseases. The need to combat these superbugs mandates the development of new drugs overcoming antibacterial resistance. Various pathogens such as *Clostridium perfringens*, *Pseudomonas aeruginosa* and *Streptococcus pneumoniae* express sialidases. These enzymes specifically catalyze the hydrolysis of terminal sialic acids from glycoconjugates (Shinya *et al.*, 2006 ▶) and play pivotal roles in the pathogenesis of a number of microbial diseases, including cholera, enterotoxaemia, gas gangrene and pneumonia (Corfield, 1992 ▶; Rood, 1998 ▶). Therefore, bacterial sialidases have recently emerged as a prominent target for the treatment of bacterial infections (Nguyen *et al.*, 2010 ▶; Woo *et al.*, 2011 ▶). The latest studies on sequelae of sepsis and septic shock have focused on understanding the roles of bacterial pathogenic factors (Rittirsch *et al.*, 2008 ▶). Moreover, studies using a sialidase deletion mutant from *P. aeruginosa* showed that bacterial sialidase acts in the initial stages of pulmonary infection by targeting glycoconjugates and biofilm production (Soong *et al.*, 2006 ▶).

Since sialidase inhibitors are substrate analogues and typically exhibit competitive kinetics, it is interesting to explore natural non-substrate mimics such as flavonoids, (oligo)­stilbenes, coumarins and diarylheptanoids (Grienke *et al.*, 2012 ▶). Flavonoids, which are polyphenolic compounds, are widespread in the plant kingdom and are known to show antibacterial and antiviral effects (Liu *et al.*, 2008 ▶; Jeong *et al.*, 2009 ▶; Grienke *et al.*, 2012 ▶). Natural flavonoids have also been reported to have latent anti-influenza and antibacterial effects against SARS (severe acute respiratory syndrome) virus, MRSA (methicillin-resistant *Staphylococcus aureus*) and Chagas disease mediated by a trypanosome (Arioka *et al.*, 2010 ▶; Cho *et al.*, 2013 ▶; Navrátilová *et al.*, 2013 ▶).

Recently, we identified the natural geranylated flavonoids diplacone, mimulone, 3′-*O*-methyldiplacone and 4′-*O*-methyldiplacone from the fruit of *Paulownia tomentosa* (empress tree) and showed that they are potent inhibitors of both cholinesterase and butyrylcholinesterase (Cho *et al.*, 2012 ▶). Here, we report that geranylated flavonoids are novel inhibitors targeting bacterial sialidase. These compounds show striking competitive inhibitory effects against sialidase from *C. perfringens* (*Cp*-NanI). Importantly, *C. perfringens* is a human pathogen causing various gastrointestinal diseases such as enterotoxaemia, gas gangrene and peritonitis.

In spite of reports of a number of structures of sialidases from bacteria (Crennell *et al.*, 1993 ▶, 1994 ▶; Gaskell *et al.*, 1995 ▶), viruses (Varghese *et al.*, 1983 ▶; Burmeister *et al.*, 1993 ▶; Crennell *et al.*, 2000 ▶) and humans (Chavas *et al.*, 2005 ▶), elucidation of the binding mode of bacterial sialidase inhibitors is limited owing to the absence of structural information (Taylor, 1996 ▶). In order to understand how natural geranylated flavonoids interact with sialidase, we determined the crystal structure of the *Cp*-NanI catalytic domain (NanI_CD_; residues 243–694) in complex with diplacone at 1.9 Å resolution. The structural information on *Cp*-NanI_CD_ and comparison with the models of human sialidases provide an insight into how the natural geranylated flavonoids can behave as antibiotics and led to the elucidation of a strategy for the design of advanced inhibitors for the treatment of infectious diseases caused by *C. perfringens*.

## 2. Methods   

### 2.1. Cloning, expression and purification   

The DNA template for *C. perfringens* sialidase (*Cp*-NanI) has NCBI reference sequence WP_011590331.1. For bacterial expression, the NanI catalytic domain without the lectin domain (NanI_CD_; residues 243–694) was amplified from *C. perfringens* genomic DNA by the polymerase chain reaction and cloned into the *Nde*I and *Xho*I restriction sites of the pET-23d expression vector (Novagen), which contains a direct C-terminal hexahistidine (His_6_) tag. *Cp*-NanI_CD_ protein was expressed in *Escherichia coli* BL21 CodonPlus (DE3) cells. The transformed cells were cultured in Luria–Bertani (LB) medium containing 100 µg ml^−1^ ampicillin at 310 K. The bacterial cells were induced at 293 K for 20 h with 0.5 m*M* isopropyl β-d-1-thiogalactopyranoside (Pharmacia) at an OD_600_ of 0.6 and then harvested by centrifugation at 4000*g* for 20 min. The harvested cells were resuspended in lysis buffer (50 m*M* sodium phosphate pH 7.0, 300 m*M* NaCl, 5 m*M* imidazole, 1 m*M* PMSF, 2 m*M* β-mercaptoethanol) and lysed by sonication, after which the lysate was centrifuged at 14 000*g* for 1 h. The resultant supernatant was applied to an immobilized metal-affinity chromatography on nickel–nitrilotriacetic acid resin (Peptron) pre-equilibrated with lysis buffer (50 m*M* sodium phosphate pH 7.0, 300 m*M* NaCl, 20 m*M* imidazole). The column was then washed with ten bed volumes of wash buffer. The His_6_-tag fused protein bound to the column was eluted with elution buffer (50 m*M* sodium phosphate pH 7.0, 300 m*M* NaCl, 300 m*M* imidazole). The samples were then purified by size-exclusion chromatography using a HiLoad 16/60 Superdex 200 column (GE Healthcare Life Science) pre-equilibrated with gel-filtration buffer (25 m*M* CHES–HCl pH 9.5, 200 m*M* NaCl), after which the fractions containing *Cp*-NanI_CD_ protein were collected. The protein was concentrated to 21.8 mg ml^−1^ using an Amicon Ultra-15 30K (Millipore) and was stored at 193 K.

### 2.2. Crystallization, data collection and structure determination   

Crystallization trials were performed using the hanging-drop vapour-diffusion method. Crystals grew at 293 K in 2 µl drops comprised of equal volumes of protein solution and reservoir solution consisting of 20%(*w*/*v*) polyethylene glycol (PEG) 3350, 0.2 *M* ammonium sulfate. The crystals were soaked in soaking solution [0.1 *M* sodium cacodylate pH 6.5, 20%(*w*/*v*) PEG 3350, 10%(*w*/*v*) glycerol, 5 m*M* diplacone, 5%(*v*/*v*) dimethyl sulfoxide] for 2 h at 293 K. The crystals were cryoprotected by transfer into cryoprotectant [20%(*w*/*v*) polyethylene glycol 4000, 10%(*v*/*v*) glycerol, 0.2 *M* ammonium sulfate] and flash-cooled in liquid nitrogen for data collection. A complete data set for *Cp*-NanI_CD_ with diplacone at 1.9 Å resolution was collected at 100 K using an ADSC Q315r detector on beamline 5C at Pohang Accelerator Laboratory (PAL) using an X-ray beam with a single wavelength (0.9795 Å). The crystal belonged to space group *P*2_1_2_1_2_1_, with unit-cell parameters *a* = 69.1, *b* = 72.6, *c* = 97.1 Å. Diffraction data were processed and scaled using the *HKL*-2000 suite (Otwinowski & Minor, 1997 ▶). The structure of *Cp*-NanI_CD_ with diplacone was determined using the molecular-replacement method with *MOLREP* (Vagin & Teplyakov, 2010 ▶) in the *CCP*4 suite (Winn *et al.*, 2011 ▶) using the apo crystal structure of *Cp*-NanI_CD_ (PDB entry 2vk5; Newstead *et al.*, 2008 ▶) as the phasing model. Automated model building and refinement of NanI_CD_ complexed with diplacone were performed with *REFMAC*5 (Murshudov *et al.*, 2011 ▶) in the *CCP*4 suite and *phenix.refine* (Adams *et al.*, 2010 ▶). *phenix.ligandfit* (Terwilliger *et al.*, 2006 ▶) was used for automated ligand building of the diplacone in *Cp*-NanI_CD_, and manual model building was performed with *Coot* (Emsley & Cowtan, 2004 ▶). Weak electron-density regions (residues 692–694) were excluded from the final structure. The Ramachandran statistics were calculated using *MolProbity* (Chen *et al.*, 2010 ▶). 96.2% of the residues were in the preferred regions and 3.8% of the residues were in allowed regions. A 2*F*_o_ − *F*_c_ composite OMIT map of diplacone contoured at 1.0σ was generated with *FFT* (Read & Schierbeek, 1988 ▶) in the *CCP*4 suite. The atomic coordinates and structure factors of *Cp*-NanI_CD_ with diplacone have been deposited in the Protein Data Bank (PDB) with accession code 4l2e. Data-collection and refinement statistics are summarized in Table 1 ▶.

### 2.3. Preparation of geranylated flavonoids   

The extraction and purification of the natural geranylated flavonoids diplacone, mimulone, 3′-*O*-methyldiplacone and 4′-*O*-methyldiplacone from *P. tomentosa* (empress tree) were performed as described previously (Cho *et al.*, 2012 ▶).

### 2.4. Enzymatic inhibitory assay   

**Figure 1:**
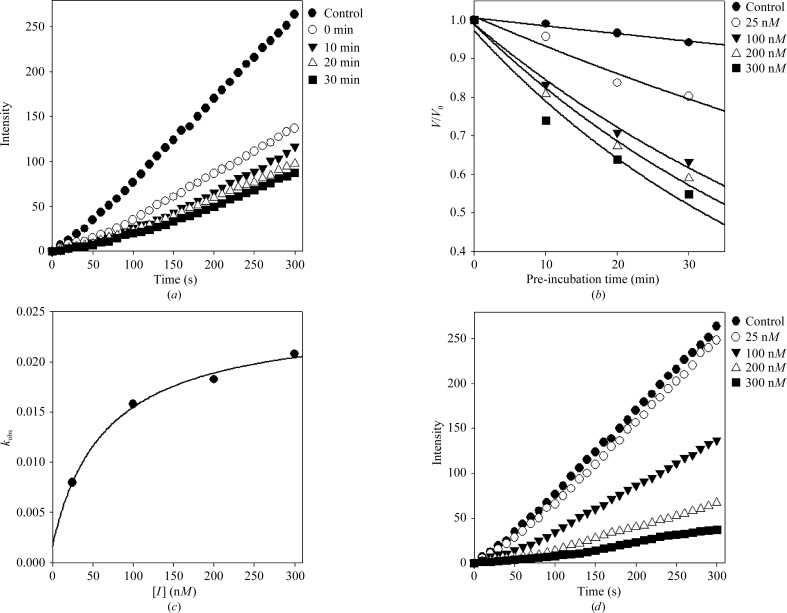
Inhibition of bacterial sialidase by diplacone. (*a*) Time-dependent inhibition of *Cp*-NanI in the presence of diplacone with varying pre-incubation times. (*b*) Pre-incubation time dependence of the fractional velocity of the enzyme-catalyzed reaction in the presence of varying concentrations of diplacone. (*c*) Dependence of *k*_obs_ on the concentration of dipacone. The *k*_obs_ values determined in (*b*) were fitted to equation (2) in Supplementary Fig. S3(*a*). (*d*) Time course of slow-binding inhibition by diplacone.

The sialidase inhibitory assay was performed as reported previously (Kim *et al.*, 2012 ▶; Cho *et al.*, 2013 ▶). Briefly, 15 µl GH33 sialidase solution (0.1 U ml^−1^) was pre-mixed with 15 µl sample solution at different concentrations in 510 µl 50 m*M* sodium acetate buffer pH 5.0 in a cuvette. 60 µl 0.125 m*M* 4-methylumbelliferyl-α-d-*N*-acetylneuraminic acid sodium salt hydrate (Sigma, catalogue No. M8639) in buffer at pH 5.0 was then added to the mixture as a substrate to start the reaction at 310 K. 4-Methylumbelliferone was immediately quantified by fluorometric determination with a SpectraMax M^2e^ Multimode Reader (Molecular Devices, USA). The excitation wavelength was 365 nm and the emission wavelength was 450 nm. Enzyme activity was recorded over a range of pre-incubation times (Fig. 1 ▶ *a*) or concentrations (Fig. 1 ▶ *b*). The data were analyzed using a nonlinear regression program (*SigmaPlot*; SPCC Inc., Chicago, Illinois, USA). The experimental enzyme activity was determined using the logistic curve represented by (1), based on a time-driven protocol with initial velocity,
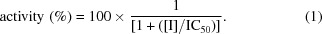



### 2.5. Homology modelling   

Model structures of human sialidases (Neu1, Neu3 and Neu4) were built using the *MODELLER* 9v7 software (Sali & Blundell, 1993 ▶). Human Neu1–4 protein sequences were retrieved from NCBI database (Neu1, NM_000434.2; Neu2, NM_005383.2; Neu3, NM_006656.5; Neu4, NM_001167599.1). On the basis of the Neu2 sequence and structure (PDB entry 1vcu; Chavas *et al.*, 2005 ▶), multiple sequence alignment and structure-based alignment were performed using *ClustalX*2 (Larkin *et al.*, 2007 ▶) and *MODELLER* 9v7 *align*2*d* (Sali & Blundell, 1993 ▶; Supplementary Fig. S1^1^). Regions from Neu1, Neu3 and Neu4 that are not conserved in the Neu2 catalytic domain (Neu1 residues 1–64, Neu3 residues 1–11, 287–300 and 315–326 and Neu4 residues 1–10, 284–336 and 355–373) were excluded. Modelling was carried out for the human Neu1, Neu3 and Neu4 catalytic domains against the chosen Neu2 template (PDB entry 1vcu) using *MODELLER* 9v7 model-single (Sali & Blundell, 1993 ▶; Supplementary Fig. S2). Each of ten outputs of the modelled structures was ranked using an internal scoring function in *MODELLER* 9v7. The reliability of the homology modelling was assessed by calculating the root-mean-square deviation (r.m.s.d.) using *PyMOL* v.1.5.0.4 (DeLano, 2004 ▶; Supplementary Table S1), evaluation of the *Z*-score using *ProSA* (Wiederstein & Sippl, 2007 ▶) and analysis of the ϕ and ψ torsion angles using *PROCHECK* (Laskowski *et al.*, 1993 ▶; Supplementary Table S2).

## 3. Results   

### 3.1. Kinetic properties of the natural geranylated *Cp*-NanI inhibitors   

In order to demonstrate that flavonoid-based natural compounds extracted from *P. tomentosa* act as inhibitors of *Cp*-NanI, a comparative activity screen was performed. This method relies on substrate hydrolysis, leading to an increase in fluorescence that can be read out in real time. Interestingly, a geranyl (3,7-dimethylocta-2,6-dienyl) group at the C6 position within the flavonoid was found to be crucial for both inhibitory potency and kinetic mechanism. For example, diplacone showed a 150-fold higher efficacy (IC_50_ = 0.11 µ*M*) than eriodictyol, a parent flavonoid lacking a geranyl group (IC_50_ = 16.4 µ*M*) (Table 2 ▶). As shown in Fig. 1 ▶, diplacone showed competitive kinetics, with a *K*_i_ value of 0.04 µ*M*. Diplacone also acted as a time-dependent inhibitor.

Slow-binding inhibition mechanisms can be investigated by pre-incubation of the enzyme with the inhibitor followed by measurement of the initial velocities of substrate hydrolysis as a function of pre-incubation time. In this way, increasing the concentration of diplacone led to a decrease in both the initial velocity (*v*_i_) and the steady-state rate (*v*_s_) (Figs. 1 ▶ *a*, 1 ▶ *b* and Supplementary Fig. S3*a*). The progress curves obtained using various concentrations of the inhibitors were fitted to equation (2) (Supplementary Fig. S3*a*) to determine *v*_i_, *v*_s_ and *k*_obs_. The results for diplacone were fitted to the a slow-binding process (equation 3; Supplementary Fig. S3*a*), illustrating a hyperbolic dependence of *k*_obs_ on the concentration of bacterial sialidase (Fig. 1 ▶ *c*). Based on the above kinetic observations, we conclude that the reaction follows a time-dependent kinetic mechanism (Fig. 1 ▶ *d*). From the results of the fit, values of 0.201 ± 0.003 and 0.0017 ± 0.004 min^−1^ were obtained for *k*_5_ and *k*_6_, respectively. Using these values derived from *k*_obs_ gives a calculated *K*_i_^app^ value of 0.065 ± 0.008 µ*M*, which is in agreement with the *K*_i_ (0.04 µ*M*) value determined using double-reciprocal plots (Lineweaver–Burk and Dixon plots; Supplementary Fig. S4). The hyperbolic dependence observed with the inhibitors suggests that diplacone inhibits sialidase by the rapid formation of an enzyme complex (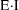) which slowly isomerizes to form a modified enzyme complex (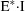) (Fig. 1 ▶ and Supplementary Fig. S3*b*).

### 3.2. Structure of *Cp*-NanI in complex with diplacone   

**Figure 2:**
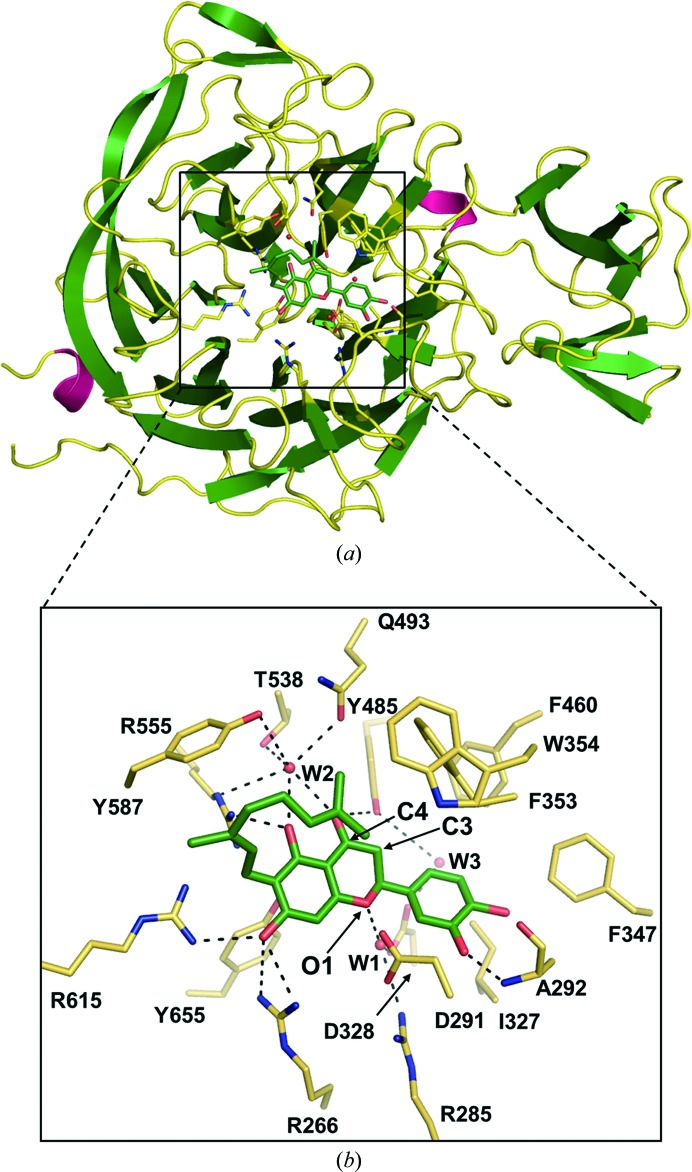
The *Cp*-NanI catalytic site with diplacone. (*a*) Overall structure of the *Cp*-NanI catalytic domain bound to diplacone. (*b*) Details of the mode of binding. Diplacone and three water molecules (W1, W2 and W3) are shown as green sticks and red spheres, respectively. Hydrogen bonds are displayed as dashed lines.

Comparison of the structures of sialidases from bacteria (Crennell *et al.*, 1993 ▶, 1994 ▶; Gaskell *et al.*, 1995 ▶), viruses (Varghese *et al.*, 1983 ▶; Burmeister *et al.*, 1993 ▶; Crennell *et al.*, 2000 ▶) and humans (Chavas *et al.*, 2005 ▶) suggested that sialidases share a highly conserved active site located within the six-bladed β-propeller fold (Supplementary Table S3 and Supplementary Fig. S5). To investigate the mode of binding of diplacone to the enzyme, we determined the structure of *Cp*-NanI in complex with diplacone. The structure of *Cp*-NanI_CD_ in complex with diplacone shows little deviation from previously published structures. The β-propeller fold within the apo structure (PDB entry 2vk5; Newstead *et al.*, 2008 ▶) and the structure of *Cp*-NanI in complex with the substrate Neu5Ac (*N*-acetyl­neuraminic acid; PDB entry 2bf6; Newstead *et al.*, 2008 ▶) show root-mean-square deviations (r.m.s.d.s) relative to our structure of 0.12 and 0.11 Å, respectively (Figs. 2 ▶ *a* and 3 ▶ *a*). Consistent with our kinetic analysis, diplacone was bound to the enzyme active site. The principal interactions between the inhibitor and enzyme were hydrogen bonds and hydrophobic interactions. The 3′,4′,5,7′-hydroxyl-4-oxo group and the O atom at the 1-position of diplacone form direct or indirect hydrogen bonds with side chains of the tri-arginyl cluster (Arg266, Arg555 and Arg615) as well as residues Arg285, Asp328, Phe353, Tyr485, Gln493, Thr538, Tyr655 and the main chain of Ala292, in addition to three water molecules (W1, W2 and W3) (Fig. 2 ▶ *b* and Supplementary Fig. S6). The flavanone backbone of diplacone also displays hydrophobic contacts with Ile327, Phe347, Phe460, Tyr485 and Tyr655, which orientate the inhibitor correctly and stabilize the bound conformation.

The crystal structure also provides an explanation for the weak inhibition shown by the parent eriodictyol and three other geranylated flavonoids (3′-*O*-methyldiplacone, 4′-*O*-methyldiplacone and mimulone) studied in this manuscript (Table 2 ▶; Fig. 2 ▶ *b*). The geranyl group made a suitable hydrophobic interaction with four aromatic residues: Phe353, Trp354, Phe460 and Tyr587. Therefore, diplacone (IC_50_ of 0.11 µ*M*) showed an approximately 150-fold higher inhibitory effect compared with eriodictyol lacking the geranyl group (IC_50_ of 16.4 µ*M*). The hydroxyl group at C3′ of diplacone also made additional hydrogen bonds with the main-chain N atom of Ala292. Introducing methylation of this position (3′-*O*-methyldiplacone, IC_50_ of 14.3 µ*M*) or the adjacent C4′ (4′-*O*-methyldiplacone, IC_50_ of 12.9 µ*M*) would disturb this hydrogen bond and cause steric hindrance with the main chain of Ala292 and the side chains of Ile327 and Phe347. On the other hand, eliminating the C3′ hydroxyl group (mimulone, IC_50_ of 6.1 µ*M*) does not lead to structural clashes with the protein but prevents hydrogen-bond formation. Therefore, maintaining the geranyl and C3′ hydroxyl group is important for the further development of diplacone-based *Cp*-NanI inhibitors.

### 3.3. Comparison with apo and substrate complex of *Cp*-NanI   

**Figure 3:**
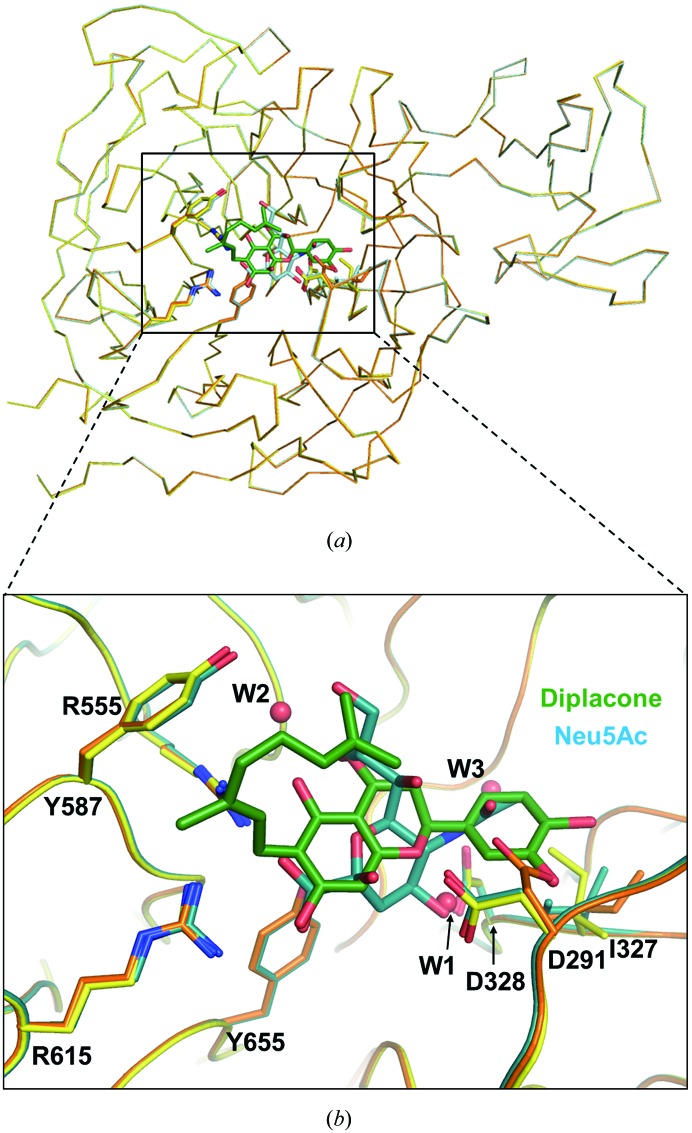
Structural comparison of apo NanI_CD_, NanI_CD_–Neu5Ac and NanI_CD_–diplacone. (*a*) Overall superposed structures of apo NanI_CD_ (orange) and the NanI_CD_–Neu5Ac (cyan) and NanI_CD_–diplacone (yellow) complexes. (*b*) Catalytic site. Diplacone and Neu5Ac molecules are displayed as green and cyan sticks, respectively. Water molecules (W1, W2 and W3) are shown as red spheres.

Although the overall structure of *Cp*-NanI_CD_ in complex with diplacone is similar to those of the apo enzyme and the complex with Neu5Ac, there are interesting structural differences between the apo enzyme and the complexes with Neu5Ac and diplacone that provide useful information regarding the development of natural geranylated flavonoid-based synthetic inhibitors (Fig. 3 ▶ *b*). Asp291 in the diplacone and Neu5Ac complexes, which is a mechanistically important acid/base catalyst (Crennell *et al.*, 1993 ▶), rotates by approximately 120° compared with the apo structure to make a hydrogen bond to the 3′-hydroxyl of diplacone or the 4′-hydroxyl of Neu5Ac. Moreover, the rotation of Asp291 also induces a conformational change of Ile327 in the apo enzyme compared with both complexes. Relative to the diplacone-bound complex, in the Neu5Ac-bound complex Ile327 moves to make a suitable hydrophobic interaction with the β-carbon of Asp291 and the 5′-acetamide moiety of the Neu5Ac molecule. Apo *Cp*-NanI_CD_ and the diplacone complex showed a hydrogen-bond interaction mediated by one water molecule (W1), the position of which nearly overlaps with the 4′-hydroxyl group of Neu5Ac. On the other hand, the hydrogen bond between Asp328 and the 4′-hydroxyl group of Neu5Ac seems to be responsible for inducing the conformational change of Ile327. *Cp*-NanI_CD_ in complex with diplacone has the same side-chain orientation of Asp291 as in the substrate (Neu5Ac) complex. In addition, an extra water molecule (W1) rather than the 4′-hydroxyl group of Neu5Ac stabilizes the diplacone complex. Therefore, together with the kinetic inhibition assay, the structure of the *Cp*-NanI_CD_–diplacone complex suggests that diplacone binds to the same substrate-binding site and acts as a competitive inhibitor.

## 4. Discussion   

We have extracted natural geranylated flavonoids from *P. tomentosa* and shown that they exhibit notable kinetic properties against sialidase from *C. perfringens*, a bacterium causing various gastrointestinal diseases. We also found that diplacone is the most effective inhibitor among these flavonoids. This inhibitor also shows a time-dependent competitive inhibition pattern. The crystal structure of the *Cp*-NanI catalytic domain in complex with diplacone rationalized how the geranyl and C3′ hydroxyl groups of diplacone contribute to the stability of the enzyme–inhibitor complex (Table 2 ▶).

From the structural information described above, we suggest further modification strategies of diplacone to improve its inhibitory properties against *C. perfringens* NanI. In the *Cp*-NanI–diplacone complex, two water molecules (W1 and W2) mediate hydrogen bonds between *Cp*-NanI residues (Arg285 and Asp328 with W1 and Gln493, Thr538 and Tyr587 with W2) of the catalytic site and diplacone atoms (O1 with W1 and C4 with W2) (Table 2 ▶ and Fig. 2 ▶ *b*). Introducing polar moieties such as carboxylic and amino groups at the O1 or C4 positions of diplacone may possibly improve binding affinity in the *Cp*-NanI catalytic site through the formation of additional hydrogen bonds. Addition of hydrophobic groups, including phenyl, benzyl and cyclohexyl groups, to C3 of diplacone may also increase the binding affinity *via* hydrophobic contact with the hydrophobic patch (Phe353, Trp354 and Phe460) of the enzyme. Furthermore, aryl groups (phenyl or benzyl) may form a potential dipole–dipole interaction with Phe353.

**Figure 4:**
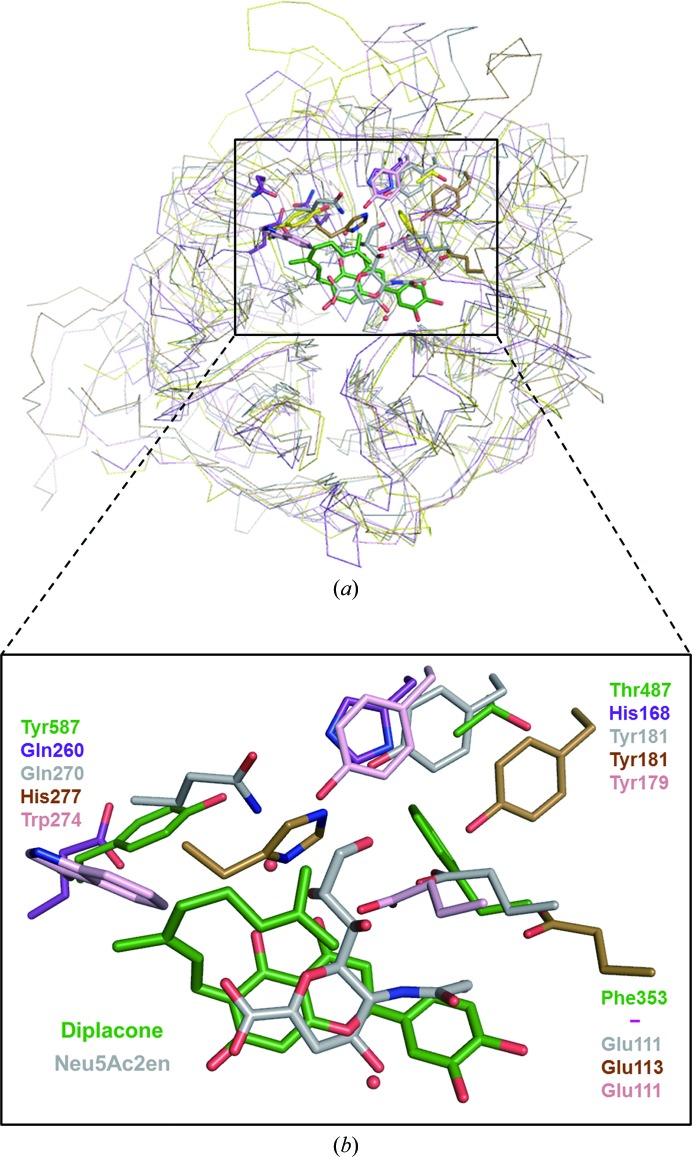
Structural comparison of *Cp*-NanI_CD_, the human Neu2–Neu5Ac2en complex structure and homology models of human Neu1, Neu3 and Neu4. (*a*) Overall superposed structures of *Cp*-NanI_CD_ (green), the human Neu2–Neu5Ac2en complex structure (grey) and homology models of human Neu1 (purple), Neu3 (brown) and Neu4 (magenta). (*b*) Detailed view. Diplacone and Neu5Ac2en molecules are displayed as green and grey sticks, respectively. Water molecules are shown as red spheres.

Because sialidases are expressed not only in *C. perfringens* but also in human lysosomes (Neu1 and Neu4), cytosol (Neu2) and membranes (Neu3), diplacone could cause undesirable effects in humans because it may inhibit human sialidases. To predict how diplacone would interact with human silalidase counterparts, we modelled human sialidases (Neu1, Neu3 and Neu4) using the previously determined Neu2 structure and compared the specificity of diplacone with *C. perfringens* and human sialidases (Fig. 4 ▶ *a* and Supplementary Fig. S2). The *Cp*-NanI catalytic domain with bound diplacone was superimposed with the crystal structure of human Neu2 with bound 2-deoxy-2,3-dehydro-*N*-acetylneuraminic acid (Neu5Ac) and models of human Neu1, Neu3 and Neu4 that were constructed using Neu2 as a template. The geranyl group of diplacone is essential for inhibition of the bacterial enzyme as it occupies a hydrophobic pocket containing Phe353, Thr487 and Tyr587. In human Neu proteins the corresponding residues are polar and would not interact favourably with the geranyl group (Fig. 4 ▶ *b*). Consequently, the structural comparison between the human sialidases Neu1–Neu4 and the *Cp*-NanI–diplacone complex suggests that the interaction between human sialidases and diplacone is likely to be unfavourable owing to polar or ionic repulsion.

On the other hand, despite extensive efforts in drug development targeting influenza neuraminidase, a series of drug-resistant mutants from influenza viruses have been reported, such as the I223R/H275Y dual mutation of neuraminidase from influenza A H1N1, which shows enormous resistance towards oseltamivir (approximately 7500 times that of the wild type; van der Vries *et al.*, 2012 ▶). To overcome drug resistance, various natural flavonoid compounds containing a flavanone backbone with inhibitory effects on viral neuraminidases (Grienke *et al.*, 2012 ▶) may be useful. Because diplacone has a flavanone backbone, structural information on the *Cp*-NanI–diplacone complex would help in the discovery of viral neuraminidase inhibitors. In this light, we compared the structure of the *Cp*-NanI_CD_–diplacone complex with two structures of drug-resistant mutants of viral neuraminidases (the I223R mutant of H1N1 and the H274Y mutant of H5N1) to examine the use of diplacone against influenza viral neuraminidases. Despite the low sequence identity and low homology of viral neuraminidases from influenza A (IA-NA) and B (IB-NA) with *Cp*-NanI_CD_ (identity: IA-NA, 11.56%; IB-NA, 12.55%; homology: IA-NA, 29.15%; IB-NA, 29.53%), their overall structures are well conserved (Supplementary Figs. S1 and S7*a*). Moreover, diplacone seems to be accessible regardless of the I223R or the H275Y mutation. The hydroxyl group at the C4 position of diplacone is able to interact with Glu276 of the H275Y mutant as in the wild type, which suggests that diplacone may be a potential lead compound for the development of inhibitors of drug-resistant mutants of viral neuraminidases (Supplementary Fig. S7*b*).

In summary, we isolated the geranylated flavonoids diplacone, mimulone and 4′-*O*-methyldiplacone from *P. tomentosa* and showed their inhibitory activities against *Cp*-NanI using comparative activity screening. The first crystal structure of sialidase with a geranylated flavonoid provides structural insights into the binding mode of natural flavonoid-based inhibitors at atomic resolution. The higher inhibitory potency of diplacone relative to the other compounds studied here can be ascribed to two principal factors. Firstly, the 3′-hydroxyl group stabilizes the enzyme–inhibitor complex through hydrogen bonding to the main chain of Ala292. Secondly, additional hydrophobic interactions are observed between the geranyl group and the hydrophobic pocket formed by Phe353, Trp354 and Tyr587 in the active site. Importantly, in the four variants of human sialidase this pocket has a significantly different polarity and would not be expected to interact favorably with the geranyl group. Glu111, Tyr179 and Tyr181 of human Neu2 are not conserved in bacterial and viral sialidases and are considered as important sites for the design of selective drugs targeting bacteria and viruses (Burmeister *et al.*, 1993 ▶). However, our biochemical and structural data suggest that the hydrophobic binding pocket of *Cp*-NanI which binds the geranyl group within diplacone may also be exploited as a means to derive selectivity for bacterial *versus* human sialidases. Our results will provide valuable information for the design of new selective antibacterial or antiviral agents using flavonoids.

## 5. Related literature   

The following references are cited in the Supporting Information: Nicholas & Deerfield (1997 ▶), Sali & Blundell (1993 ▶), Chavas *et al.* (2005 ▶), Newstead *et al.* (2008 ▶), Luo *et al.* (1998 ▶), Telford *et al.* (2011 ▶), Buschiazzo *et al.* (2000 ▶), Crennell *et al.* (1996 ▶), Xu *et al.* (2008 ▶) and Varghese *et al.* (1995 ▶).

## Supplementary Material

PDB reference: *Cp*-NanI catalytic domain, complex with diplacone, 4l2e

Supporting Information.. DOI: 10.1107/S1399004714002971/mv5098sup1.pdf

## Figures and Tables

**Table 1:** Data-collection and refinement statistics Values in parentheses are for the highest resolution shell.

Data collection	
X-ray source	Beamline 5C, PAL
Wavelength (Å)	0.9795
Resolution range (Å)	50–1.9 (1.93–1.90)
Space group	*P*2_1_2_1_2_1_
Unit-cell parameters (Å)	*a* = 69.1, *b* = 72.6, *c* = 97.1
No. of observed reflections	195578
No. of unique reflections	39033
Completeness (%)	99.1 (98.1)
*R*_merge_ † (%)	5.5 (9.6)
Mean *I*/σ(*I*)	32.7 (26.9)
Multiplicity	5.0 (5.1)
Refinement statistics	
Resolution range (Å)	40–1.9
*R*_work_/*R*_free_ ‡ (%)	15.8/19.4
No. of atoms	
Protein	3526
Diplacone	31
Ca^2+^	2
Water	574
Average *B* factors (Å^2^)	
Protein	31.2
Diplacone	50.1
Ca^2+^	36.4
Water	41.7
R.m.s. deviations from ideal geometry	
Bond lengths (Å)	0.012
Bond angles (°)	1.30
Ramachandran plot	
Most favoured regions (%)	96.2
Allowed regions (%)	3.8
PDB code	4l2e

† *R*_merge_ = 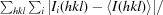 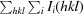, where *I*(*hkl*) is the intensity of reflection *hkl*, 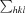 is the sum over all reflections and 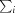 is the sum over *i* measurements of reflection *hkl*.

‡ *R*_work_ = 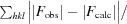 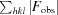
|; *R*_free_ is the *R* value calculated for 5% of the data set that was not included in the refinement.

**Table 2:** Structures and inhibitory activities (IC_50_) of flavonoid-based *Cp*-NanI inhibitors

Compound†	Structure	IC_50_ (µ*M*)
Eriodictyol	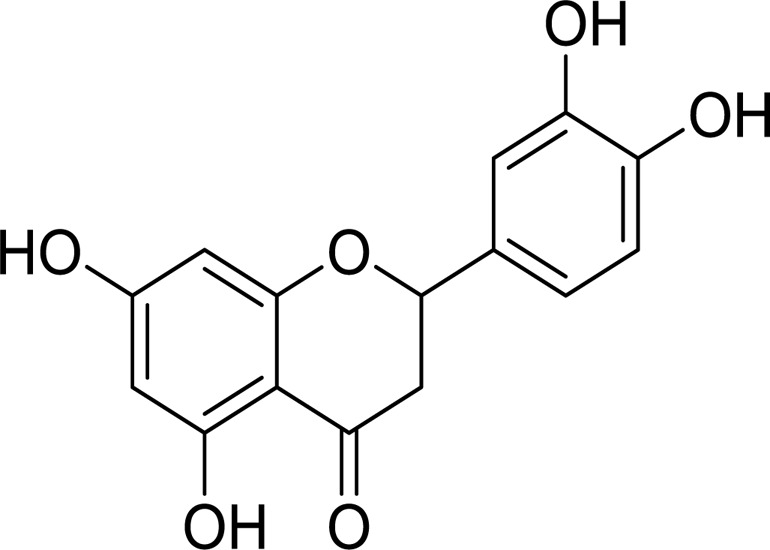	16.4
3′-*O*-Methyldiplacone	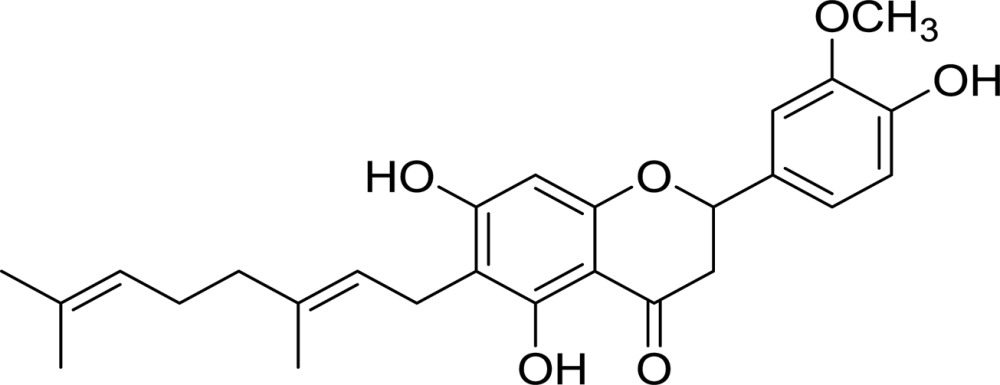	14.3
4′-*O*-Methyldiplacone	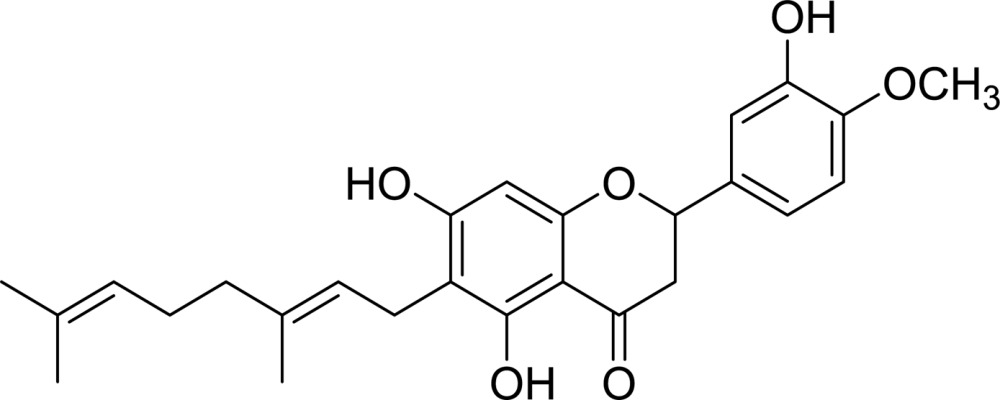	12.9
Mimulone	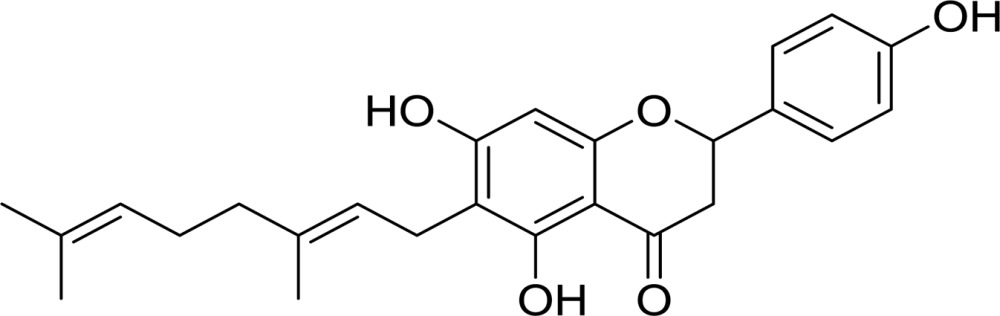	6.1
Diplacone	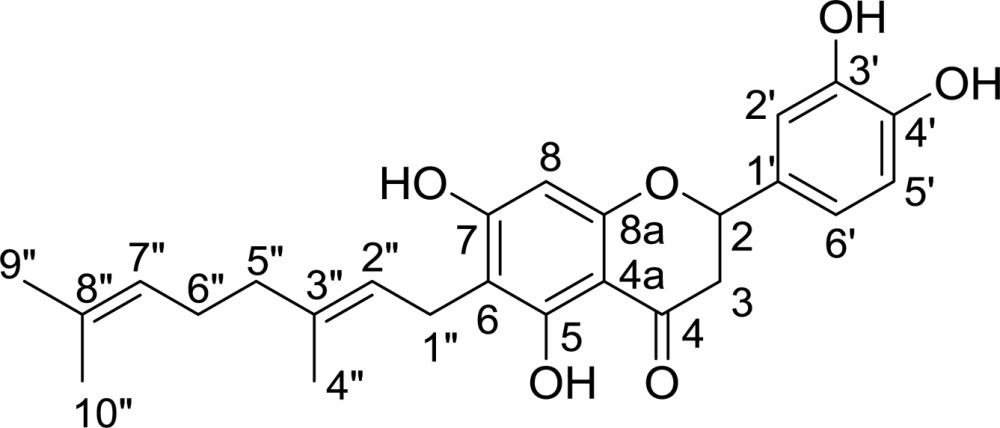	0.110

† IUPAC nomenclature: eriodictyol, (2*S*)-2-(3,4-dihydroxyphenyl)-5,7-dihydroxy-4-chroman-one; 3′-*O*-methyldiplacone, 6-[(2*E*)-3,7-dimethylocta-2,6-dienyl]-5,7-dihydroxy-2-(3-hydroxy-4-methoxyphenyl)-2,3-dihydrochromen-4-one; 4′-*O*-methyldiplacone, 6-[(2*E*)-3,7-dimethylocta-2,6-dienyl]-5,7-dihydroxy-2-(3-hydroxy-4-methoxyphenyl)-2,3-dihydrochromen-4-one; mimulone, (2*S*)-6-[(2*E*)-3,7-dimethylocta-2,6-dienyl]-5,7-dihydroxy-2-(4-hydroxyphenyl)-2,3-dihydrochromen-4-one; diplacone, 2-(3,4-dihydroxyphenyl)-6-[(2*E*)-3,7-dimethylocta-2,6-dienyl]-5,7-dihydroxy-2,3-dihydrochromen-4-one.

## Footnotes

1 Supporting information has been deposited in the IUCr electronic archive (Reference: MV5098).
