# Structural basis of sialidase in complex with geranylated flavonoids as potent natural inhibitors. Corrigendum

**DOI:** 10.1107/S1399004715000899

**Published:** 2015-02-03

**Authors:** Youngjin Lee, Young Bae Ryu, Hyung-Seop Youn, Jung Keun Cho, Young Min Kim, Ji-Young Park, Woo Song Lee, Ki Hun Park, Soo Hyun Eom

**Affiliations:** a School of Life Sciences, Gwangju Institute of Science and Technology (GIST), Buk-gu, Gwangju 500-712, Republic of Korea; b Steitz Center for Structural Biology, Gwangju Institute of Science and Technology (GIST), Buk-gu, Gwangju 500-712, Republic of Korea; c Department of Chemistry, Gwangju Institute of Science and Technology (GIST), Buk-gu, Gwangju 500-712, Republic of Korea; d Infection Control Research Center, Korea Research Institute of Bioscience and Biotechnology, Jeongeup 580-185, Republic of Korea; e Division of Applied Life Science (BK21 Program, IALS), Graduate School of Gyeongsang National University, Jinju 660-701, Republic of Korea

**Keywords:** sialidase, NanI, geranylated flavonoid, diplacone, sialidase inhibitor, corrigendum

## Abstract

A correction is made to the article by Lee *et al.* [(2014) *Acta Cryst.* D**70**, 1357–1365].

## Abstract 2

The article by Lee *et al.* [(2014) *Acta Cryst.* D**70**, 1357–1365] is corrected.

The authors of Lee *et al.* (2014 ▶) have made a careful re-analysis of all of data sets used in their structure determination of the putative complex between the human pathogen *Clostridum perfringens* sialidase and the natural inhibitory compound, geranylated flavonoid diplacone, described in the article. They now no longer believe that their structure (PDB accession number: 4l2e) is supported by X-ray diffraction data and thus have asked for the 4l2e entry to be made obsolete in the Protein Data Bank. This does not affect the validity of the biochemical characterization of specific interactions of the sialidase with diplacone and other naturally occurring compounds reported in the paper.
