# Supplementary material for: Structural basis of sialidase in complex with geranylated flavonoids as potent natural inhibitors
Source: Acta Crystallogr D Biol Crystallogr. 2014 Apr 30;70(Pt 5):1357–65. doi: 10.1107/S1399004714002971 (PMC4014123; doi:10.1107/S1399004714002971)
Supplement: Supplementary file 1 [file d-70-01357-sup1.pdf]

Supplementary Information

Structural Basis of Sialidase in Complex with Geranylated Flavonoids as Potent Natural Inhibitors

Youngjin Lee,[a,b‡] Young Bae Ryu,[d‡] Hyung-Seop Youn,[a,b‡] Jung Keun Cho,[e] Young Min Kim,[d] Ji-Young Park,[d] Woo Song Lee,[d] Ki Hun Park[e*] and Soo Hyun Eom[a,b,c*]

[a]School of Life Sciences, [b]Steitz Center for Structural Biology and [c]Department of Chemistry, Gwangju Institute of Science and Technology (GIST), Buk-gu, Gwangju 500-712, Republic of Korea, [d]Infection Control Research Center, Korea Research Institute of Bioscience and Biotechnology, Jeongeup 580-185, Republic of Korea, and [e]Division of Applied Life Science (BK21 plus, IALS), Graduate School of Gyeongsang National University, Jinju 660-701, Republic of Korea

[‡] These authors contributed equally to this work.
Correspondence email: khpark@gnu.ac.kr; eom@gist.ac.kr

[*]Corresponding authors:

Ki Hun Park: khpark@gnu.ac.kr, Phone : +82- 55-751-5472, Fax: + 82-55-757-0178

Soo Hyun Eom[#]: eom@gist.ac.kr, Phone: +82-62-715-2519, Fax: +82-62-715-2521
Person to contact for this submission

1. Supplementary Figures

Supplementary Figure 1. Multiple sequence alignment[1] of *Clostridium perfringens (Cp)*-NanI and human (*Homo sapiens* (*Hs*)) Neu1–4. 3

Supplementary Figure 2. Superposed overall structures of *Hs*-Neu1–4. 5

Supplementary Figure 3. Scheme for time-dependent enzyme inhibition. 6

Supplementary Figure 4. Lineweaver-Burk plot for inhibition of *Cp*-NanI by diplacone. 7

Supplementary Figure 5. Superposition of the eight sialidase catalytic domains. 8

Supplementary Figure 6. $2F_o - F_c$ composite omit map of diplacone contoured at 1.0 σ. 9

Supplementary Figure 7. Structural comparison of *Cp*-NanI and drug-resistant mutants of viral neuraminidases. 10

2. Supplementary Tables

Supplementary Table 1. Root mean square deviation (Å) of *Hs*-Neu1–4. 11

Supplementary Table 2. Validation results of *Hs*-Neu1–4 structures. 12

Supplementary Table 3. Conservation of the structures of sialidase catalytic domains. 13

3. Supplementary References 14

a

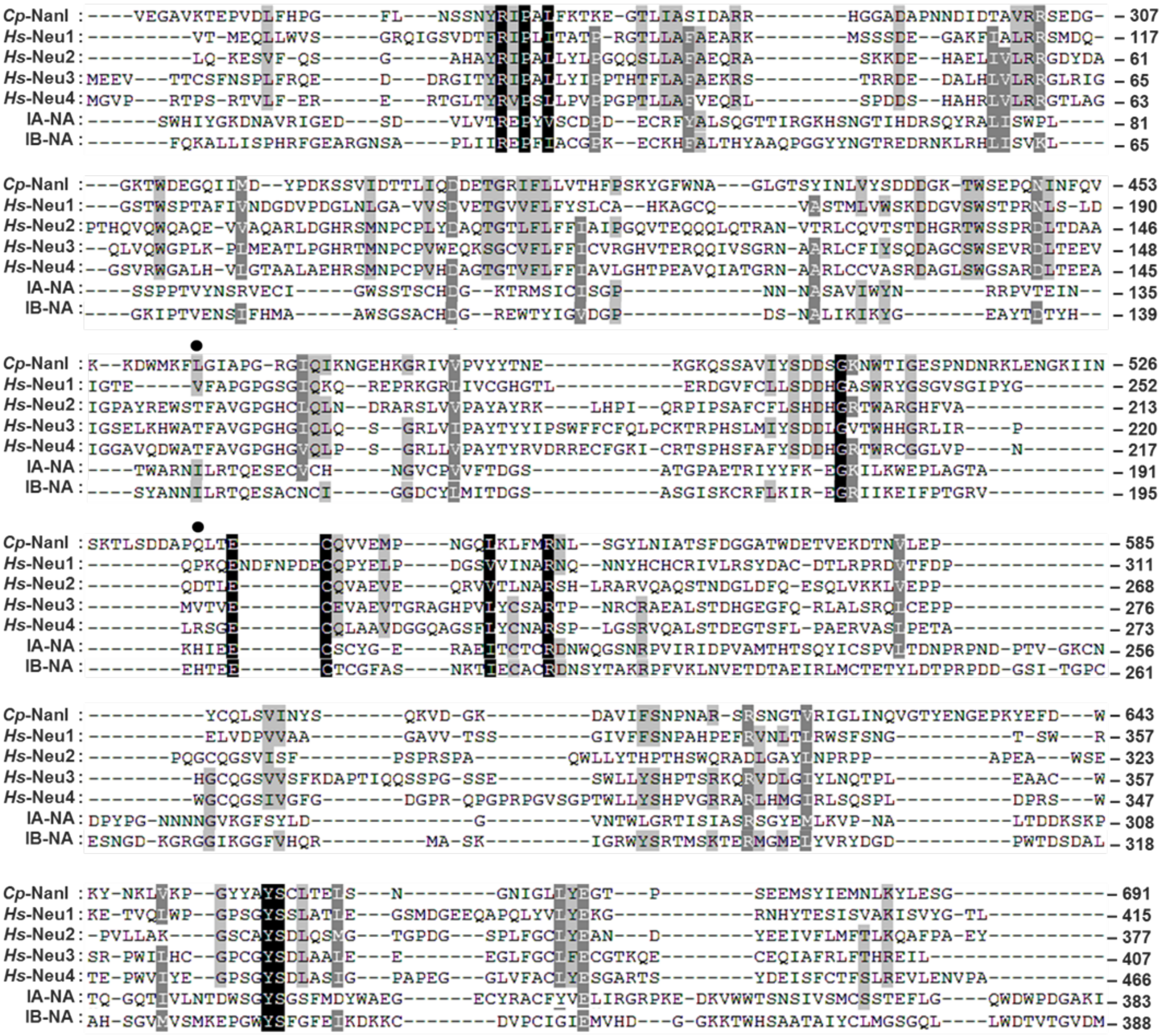

b

Proteins	Conserved residues						Variable residues
Cp-NanI	R266	D328	E539	R555	R615	Y655	F353, T487, Y587
Hs-Neu1	R26	—	E205	R228	R289	Y318	E111, Y179, W274
Hs-Neu2	R21	N86	E218	R237	R304	Y334	E111, Y181, Q270
Hs-Neu3	R25	N88	E225	R245	R315	Y345	E113, Y181, H277
Hs-Neu4	R23	N86	E222	R242	R313	Y343	H168, Q204, Q230, Q260
IA-NA	R119	—	E279	R294	R372	Y406	R155, I224, N296
IB-NA	R115	—	E275	R291	R373	Y108	R149, I220, N293

Supplementary Figure 1. Multiple sequence alignment[1] of *Clostridium perfringens (Cp)*-NanI and human (*Homo sapiens* (*Hs*)) Neu1–4. **(a)** Total five sequences were aligned: *Cp*-NanI (*WP_011590331.1*, residues 243–691 and Δ361–426); *Hs*-Neu1 (*NM_000434.2*, residues 65–407); *Hs*-Neu2 (*NM_005383.2*, residues 12–377); *Hs*-Neu3 (*NM_006656.5*, residues 12–407, Δ287–300 and Δ315–326); *Hs*-Neu4 (*NM_001167599.1*, residues 11–466, Δ284–336 and Δ355–373); Influenza A neuraminidase (IA-NA) (strain A/Tern/Australia/G70C/1975 H11N9) (M17813.1, residues, 14−383); Influenza B neuraminidase (IB-NA) (strain B/Beijing/1/1987) (M54967.2, residues, 16−388). The italic codes mean NCBI GenBank reference sequence numbers. Residues showing multi-drug resistances of viral NAs are shown as black filled circles in the above of the sequences. **(b)** Conserved and variable residues of *Cp*-NanI, *Hs*-Neu1–4, IA-NA, and IB-NA.

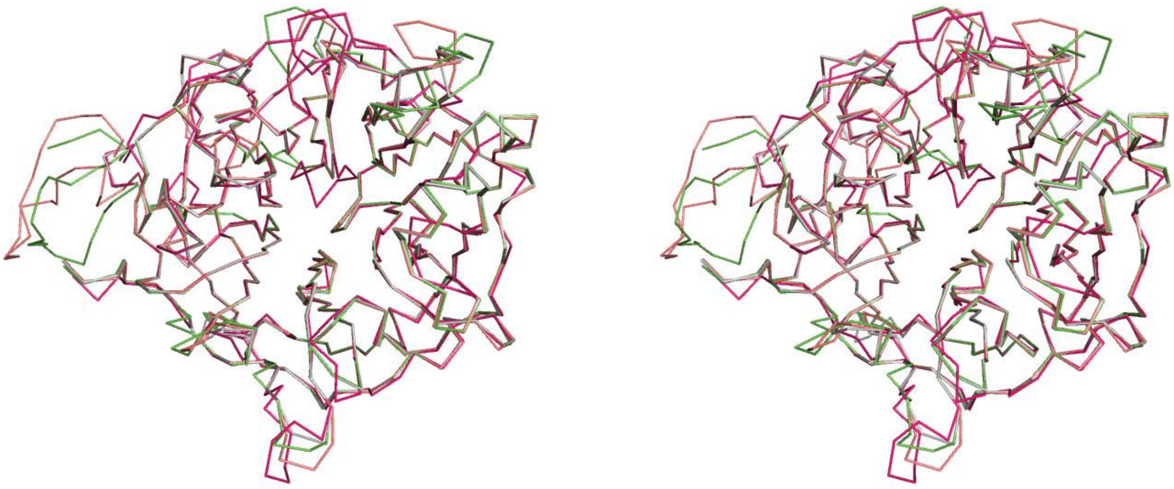

Supplementary Figure 2. Superposed overall structures of *Hs*-Neu1–4. *Hs*-Neu1 (purple), *Hs*-Neu3 (green) and *Hs*-Neu4 (magenta) was built from Modeller9v7 software[2] based on reported *Hs*-Neu2 structure (PDB code, 1VCU; grey) [3].

a

$$[P] = v_{st} + (v_i - v_s)\,[1 - \exp(-k_{obs}t)] / k_{obs} \qquad (2)$$

$$k_{obs} = k_6 + [(k_5 \times [I]) / (K_i^{app} + [I])] \qquad (3)$$

b

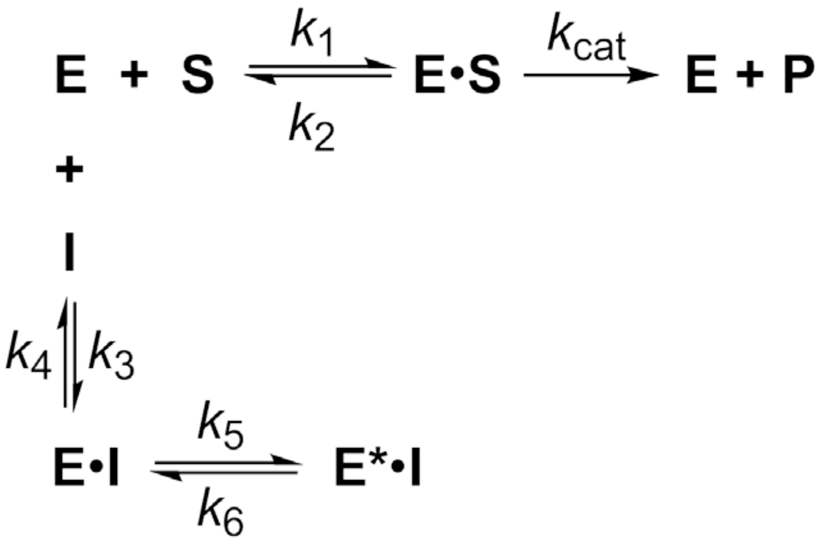

Supplementary Figure 3. Scheme for time-dependent enzyme inhibition. **(a)** Equations, (2) and (3) to determine v_i, v_s, and k_{obs} from the curves using various concentrations of the inhibitors. **(b)** The upper part denotes the turnover of the enzyme in the absence of inhibition. The lower part illustrates the equilibrium for a slow-binding inhibition process.

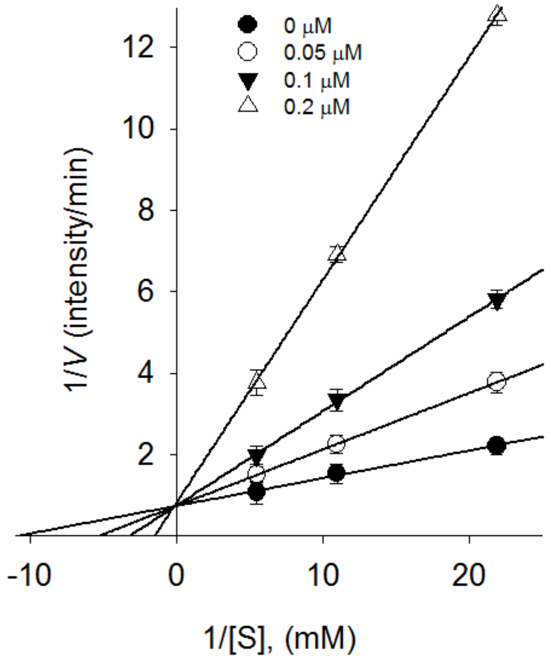

Supplementary Figure 4. Lineweaver-Burk plot for inhibition of *Cp*-NanI by diplacone.

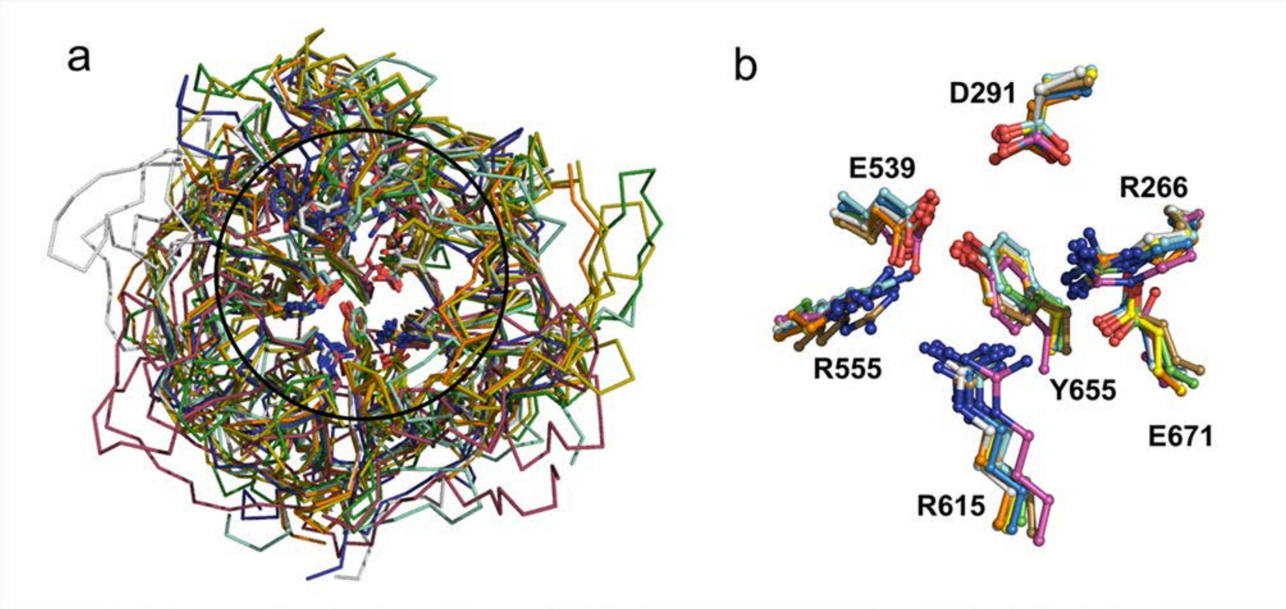

Supplementary Figure 5. Superposition of the eight sialidase catalytic domains. **(a)** Superposed sialidase catalytic domains (2VK5, grey; 1SLL, orange; 2XCY, blue; 1MZ5, green; 2SIL, brown; 2VW0, yellow; 7NN9, magenta; 1VCU, blue). Detail information was described in Table S1. **(b)** Structural conservation of the residues in the active site. The residues were labeled based on *Cp*-NanI sequence.

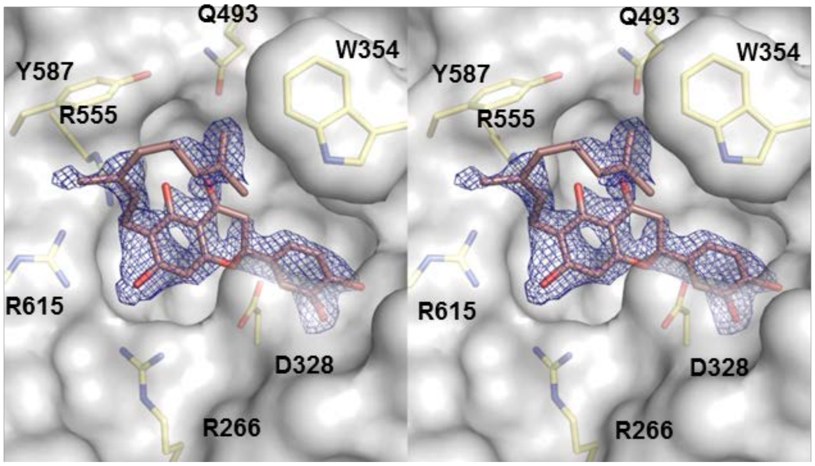

Supplementary Figure 6. $2F_o - F_c$ composite omit map of diplacone contoured at 1.0 σ.

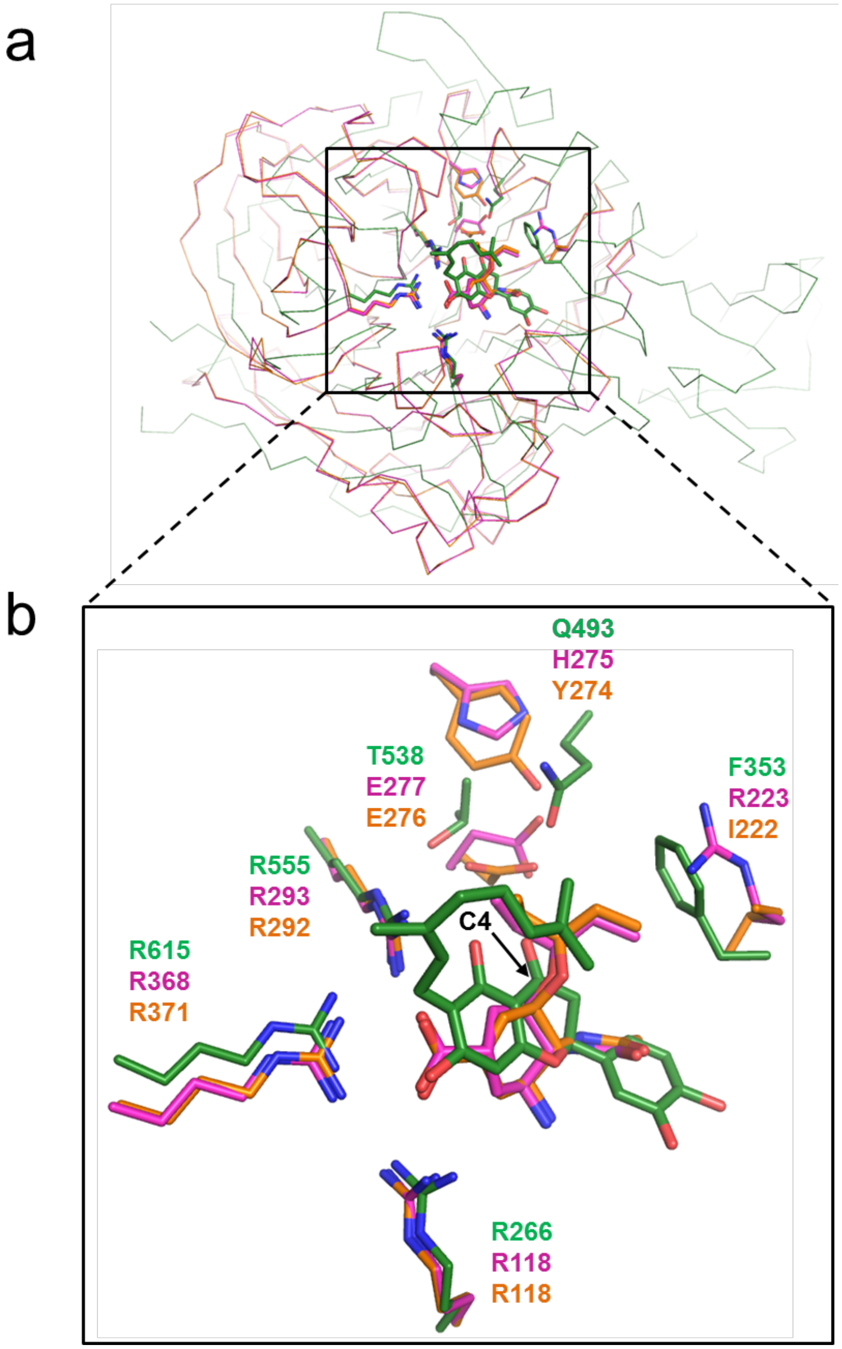

Supplementary Figure 7. Structural comparison of *Cp*-NanI and drug-resistant mutants of viral neuraminidases. *Cp*-NanI-diplacone, H1N1 viral NA (I223R)-oseltamivir (PDB code, 4B7J), and H5N1 viral NA (H274Y)-oseltamivir (PDB code, 3CL0) complexes are colored in green, magenta, and orange, respectively. **(a)** Overall superposed structures. **(b)** Detail view of the active sites.

Supplementary Table 1. Root mean square deviation (Å) of *Hs*-Neu1–4.

**Proteins**	***Hs*-Neu1**[§]	***Hs*-Neu2**[*]	***Hs*-Neu3**[§]	***Hs*-Neu4**[§]
***Hs*-Neu1**[§]	–	0.356	0.481	0.317
***Hs*-Neu2**[*]	–	–	0.134	0.120
***Hs*-Neu3**[§]	–	–	–	0.156

[*] Chain A of *Hs*-Neu2 (PDB code, 1VCU)
[§] *Hs*-Neu1, *Hs*-Neu3 and *Hs*-Neu4 were built by homology modeling based on *Hs*-Neu2.

Supplementary Table 2. Validation results of *Hs*-Neu1–4 structures.

	***Hs*-Neu1**§	***Hs*-Neu2***	***Hs*-Neu3**§	***Hs*-Neu4**§
**Ramachandran plot**				
**Most favored (%)**	81.0	77.7	81.6	86.6
**Additionally allowed (%)**	14.8	22.3	16.9	10.6
**Generously allowed (%)**	2.0	0	1.5	0.9
**Disallowed (%)**	2.3	0	0	1.9
**Z score**	−2.94	−5.80	−5.59	−5.04

*Chain A of *Hs*-Neu2 (PDB code, 1VCU)

§ *Hs*-Neu1, *Hs*-Neu3 and *Hs*-Neu4 were built by homology modeling based on *Hs*-Neu2.

Supplementary Table 3. Conservation of the structures of sialidase catalytic domains.

Gene name	Species	PDB code	RMSD (Å)
NanI	*Clostridium perfringens*	2VK5[4]	–
T7	*Macrobdella decora*	1SLL[5]	1.00
AFUA_4G 13800	*Aspergillus furmigatus*	2XCY[6]	1.15
mndE'	*Tripanosoma rangeli*	1MZ5[7]	1.30
NanH	*Salmonella typhimurium*	2SIL[8]	1.34
NanB	*Streptococcus pneumoniae*	2VW0[9]	1.20
NA	Influenza A	7NN9[10]	1.00
NEU2	*Homo sapiens*	1VCU[3]	1.19

Supplementary References

1. Nicholas, H. B. & Deerfield II, D. W. (1997). *EMBNEW.NEWS.* **4**, 14.
2. Sali, A. & Blundell, T. L. (1993). *J. Mol. Biol.* **234**, 779–815.
3. Chavas, L. M., Tringali, C., Fusi, P., Venerando, B., Tettamanti, G., Kato, R., Monti, E. & Wakatsuki, S. (2005). *J. Biol. Chem.* **7**, 469–475.
4. Newstead, S. L., Potter, J. A., Wilson, J. C., Xu, G., C.-H., Chien, Watts, A. G., Withers, S. G. & Taylor, G. L. (2008). *J. Biol. Chem.* **283**, 9080–9088.
5. Luo, Y., Li, S. C., Chou, M. Y., Li, Y. T. & Luo, M. (1998). *Structure,* **6**, 521–530.
6. Telford, J. C., Yeung, J. H., Xu, G., Kiefel, M. J., Watts, A. G., Hader, S., Chan, J., Bennet, A. J., Moore, M. M. & Taylor, G. L. (2011). *J. Biol. Chem.* **286**, 10783–10792.
7. Buschiazzo, A., Tavares, G. A., Campetella, O., Spinelli, S., Cremona, M. L., París, G., Amaya, M. F., Frasch, A. C. & Alzari, P. M. (2000). *EMBO J.* **19**, 16–24.
8. Crennell, S. J., Garman, E. F., Philippon, C., Vasella, A., Laver, W. G., Vimr, E. R. & Taylor, G. L., (1996). *J. Mol. Biol.* **259**, 264–280.
9. Xu, G., Potter, J. A., Russell, R. J., Oggioni, M. R., Andrew, P. W., & Taylor, G. L. (2008). *J. Mol. Biol.* **384**, 436–449.
10. Varghese, J. N., Epa, V. C., & Colman, P. M. (1995). *Protein Sci.* **4**, 1081–1087.
